# Lipid and metabolic syndrome traits in coronary artery disease: a Mendelian randomization study

**DOI:** 10.1194/jlr.P120001000

**Published:** 2021-02-06

**Authors:** David G. Thomas, Ying Wei, Alan R. Tall

**Affiliations:** 1 Department of Medicine, New York Presbyterian Hospital/Weill Cornell Medicine, New York, NY, USA; 2 Department of Biostatistics, Columbia University, New York, NY, USA; 3 Division of Molecular Medicine, Department of Medicine, Columbia University, New York, NY, USA

**Keywords:** human genetics, lipoproteins, low density lipoprotein, high density lipoprotein, triglycerides, IVW, inverse variance-weighted, MR, Mendelian randomization, MR-PRESSO, Mendelian randomization-pleiotropy residual sum and outlier, OR, odds ratio, SBP, systolic blood pressure, TRL, triglyceride-rich lipoprotein, UKBB, UK Biobank

## Abstract

Mendelian randomization (MR) of lipid traits in CAD has provided evidence for causal associations of LDL-C and TGs in CAD, but many lipid trait genetic variants have pleiotropic effects on other cardiovascular risk factors that may bias MR associations. The goal of this study was to evaluate pleiotropic effects of lipid trait genetic variants and to account for these effects in MR of lipid traits in CAD. We performed multivariable MR using inverse variance-weighted and MR-Egger methods in large (n ≥ 300,000) GWAS datasets. We found that 30% of lipid trait genetic variants have effects on metabolic syndrome traits, including BMI, T2D, and systolic blood pressure (SBP). Nonetheless, in multivariable MR analysis, LDL-C, HDL-C, TGs, BMI, T2D, and SBP are independently associated with CAD, and each of these associations is robust to adjustment for directional pleiotropy. MR at loci linked to direct effects on HDL-C and TGs suggests locus- and mechanism-specific causal effects of these factors on CAD.

Mendelian randomization (MR) is a method used to infer the causal effect of a risk factor, or exposure, on a disease outcome (1). In MR, genetic variants affecting the exposure are considered to confer a randomized allocation of the exposure (2). The causal effect of changes in the exposure can then be assessed by comparing the disease outcome across genotypes. Typically, SNPs are used for MR, and their individual effects on exposures and outcomes can be collected from GWASs (3). However, in cases where exposure SNPs have pleiotropic effects on unmeasured traits, risk factors other than the exposure may be imbalanced in the groups selected by allocation of the exposure SNPs, leading to biased causal estimates in MR (2, 4).

MR has been used to assess the role of the lipid traits LDL-C, HDL-C, and TGs in CAD. SNPs at specific loci modifying LDL-C, HDL-C, and TGs have been used in MR (5, 6, 7), but lipid trait SNPs often modify more than one lipid trait because they affect enzymes or disease states, such as insulin resistance, that control the remodeling of multiple lipoproteins. Multivariable MR has been used to adjust for effects of lipid trait SNPs on multiple lipid traits while integrating data from hundreds of SNPs (8, 9). MR methods that account for measurement error in GWAS effect estimates, such as inverse-variance weighting [MR-inverse variance-weighted (IVW)], and methods that estimate bias due to pleiotropy statistically, such as MR-Egger, have been developed and applied to MR of lipid traits in CAD (4, 10, 11).

In surveys of many GWASs, lipid traits share genetic determinants with other metabolic traits, such as BMI and waist-hip ratio, which are also risk factors for CAD (12, 13, 14, 15). Derangements of lipid and other metabolic traits co-occur in the metabolic syndrome, a clinical entity comprising abdominal obesity, dyslipidemia, hypertension, and insulin resistance (16). A few studies have attempted to adjust for the impact of metabolic syndrome trait pleiotropy in lipid trait MR by omitting BMI and blood pressure SNPs or regressing on residuals after adjustment for BMI and systolic blood pressure (SBP) (17, 18). These studies have concluded that there is a causal relationship of LDL-C and TGs with CAD and have arrived at conflicting results with regard to the causal effect of HDL-C. MR-Egger analysis suggests that the apparent causal effects of both HDL-C and TGs in CAD are mediated, at least in part, by pleiotropic effects of lipid trait SNPs on other traits (9, 19); however, MR-Egger is known to have reduced power to detect causal effects compared with MR-IVW (4).

The availability of MR datasets of increasing size, including measurements of potential pleiotropic factors such as BMI, T2D, and SBP, provides the opportunity to re-address and adjust for the impact of pleiotropy in the association of lipid and metabolic syndrome traits with CAD. Using expanded datasets 2- to 6-fold larger than those previously used for pleiotropy adjustment in lipid trait MR, we found that 30% of lipid trait SNPs have pleiotropic effects on the metabolic syndrome traits BMI, SBP, or T2D. We collected lipid and metabolic syndrome trait SNPs from large (n ≥ 300,000) GWASs and modeled the effects of LDL-C, HDL-C, TGs, BMI, T2D, and SBP on CAD in multivariable MR. We were also able to assess the impact of SNPs that directly modify CAD. We found that each of the studied lipid and metabolic syndrome traits is independently associated with CAD by MR. Nonetheless, MR analysis of representative SNPs at loci with direct effects on HDL-C and TGs suggest that that the causal association between these traits and CAD is locus- and mechanism-specific.

## Materials and methods

### Ethical review

All analyses were performed on anonymized summary statistics from published GWASs with appropriate institutional review board approval (20, 21, 22, 23, 24). Separate institutional review board approval was not required for this study.

### Datasets

Lipid trait summary statistics were obtained from the Million Veteran Program GWAS (n = 291,746) (20). Lipid trait effect sizes are in SD from inverse normal transformed residuals of lipids after adjusting for age, sex, and 10 principal components of ancestry as described in Klarin et al. (20). BMI summary statistics were obtained from a meta-analysis of GIANT and UK Biobank (UKBB) datasets (n = 806,834) (21). BMI effect sizes are in SD from inverse normal transformed residuals of BMI adjusted for age, sex, recruitment center, genotyping batches, and 10 principal components as described in Pulit et al. (21). T2D summary statistics were obtained from a meta-analysis of DIAGRAM and UKBB datasets (n = 71,124 cases and 824,006 controls) (22). T2D effect sizes are expressed as log odds ratio (OR) adjusted for age, sex, array, and six principal components as described in Mahajan et al. (22). SBP summary statistics were obtained from a meta-analysis of ICBP and UKBB datasets (n = 757,601) (23). SBP effect sizes are in millimeters of mercury (untransformed) adjusted for age, sex, genotyping array, and 10 principal components as described in Evangelou et al. (23). CAD summary statistics were obtained from a meta-analysis of CARDIoGRAMplusC4D and UKBB datasets (n = 122,733 cases and 424,528 controls) (24). CAD effect sizes are expressed as log OR and adjusted for age, sex, and up to 30 principal components as described in van der Harst and Verweij (24).

### Selection of instrumental variables

We used a threshold for genome-wide significance of 1 × 10^−8^ to account for the presence of both common (minor allele frequency > 5%) and low-frequency variants (5% > minor allele frequency > 1%) and the large number of imputed SNPs (25, 26, 27). We selected genome-wide significant SNPs (*P* < 1 × 10^−8^) for each trait and used the TwoSampleMR R package (28) to clump SNPs that are in linkage disequilibrium (*R*^2^ > 0.01) within a 10 Mb interval. For locus-specific MR, we considered distinct genome-wide significant SNPs within a 1 Mb interval of the transcription start site of the selected gene.

### MR

For multivariable MR, we oriented the SNPs with respect to a positive effect on the exposure of interest as described (11) and modeled the effects of lipid traits or lipid and metabolic syndrome traits for each SNP. We considered two types of multivariable MR analysis. One is multivariable MR-IVW (10), and the second is multivariable MR-Egger (11). In both approaches, we used linear regression to model log OR for CAD against lipid or lipid and metabolic syndrome trait effects and weighted each SNP by the inverse of the variance of its log OR for the outcome. In MR-IVW, we set the intercept at zero, while in MR-Egger, the intercept is included in the model and estimated in the regression (4). The inclusion of the intercept allows the SNPs to have a direct effect on CAD without mediation through the modeled exposures but may decrease statistical power when there is no unmeasured pleiotropic effect (4). For univariable MR at selected loci, we applied univariate MR-IVW on the set of SNPs at the selected locus (29). For single-SNP MR, we constructed 95% CIs for univariable MR on individual SNPs as described (30).

### Analysis of heterogeneity

For each model, we report multivariable I^2^ based on multivariable H^2^ as described in Jackson, White, and Riley (31), which measures the between-study heterogeneity in meta-analyses, and categorized I^2^ as low, moderate, or high based on the reference values suggested by Higgins et al. (32). We used MR-pleiotropy residual sum and outlier (MR-PRESSO) (33) to identify SNPs with large inconsistent CAD effects suggestive of direct effects on CAD and considered a SNP to be pleiotropic if the multivariable MR-PRESSO test *P* was <0.05. We considered multivariable MR models with or without exclusion of SNPs demonstrating evidence of pleiotropy by MR-PRESSO in the indicated analyses to produce more robust estimates.

### Interpretation of results

For pleiotropic effects, we used a Bonferroni-corrected significance threshold of 0.05/2338 = 2 × 10^−5^. For MR analysis, we considered the results significant at a threshold of 0.05 for univariable MR, 0.05/3 = 0.0167 for three-exposure multivariable MR, and 0.05/6 = 0.0083 for six-exposure multivariable MR, reflecting Bonferroni correction for multiple testing in the setting of modeling three and six associations, respectively.

## Results

### Pleiotropic effects of lipid trait SNPs on metabolic syndrome traits

We extracted independent genome-wide significant lipid trait SNPs from the Million Veteran Program GWAS of approximately 300,000 individuals (20). We obtained 164 LDL-C SNPs, 202 HDL-C SNPs, and 179 TG SNPs at a significance level of *P* < 1 × 10^−8^, representing 343 distinct loci with available CAD data. We compared lipid trait effect sizes at these SNPs to corresponding estimates from the earlier Global Lipid Genetics Consortium GWAS (n = 188,577) (34) and found that effect estimates from these two datasets are highly concordant, although estimates from the Million Veteran Program GWAS are slightly smaller in magnitude on average (Supplemental Fig. S1), likely due to attenuation of upward bias due to the winner’s curse effect (35). Effects of lipid trait SNPs on BMI, T2D, and SBP were collected from large (n > 700,000) densely imputed meta-analyses of UKBB data with GIANT, ICBP, and DIAGRAM consortium datasets, respectively (21, 22, 23). We also assessed independent genome-wide significant SNPs for metabolic syndrome traits and obtained 897 SNPs for BMI, 207 SNPs for T2D, and 689 SNPs for SBP.

**Fig. 1:**
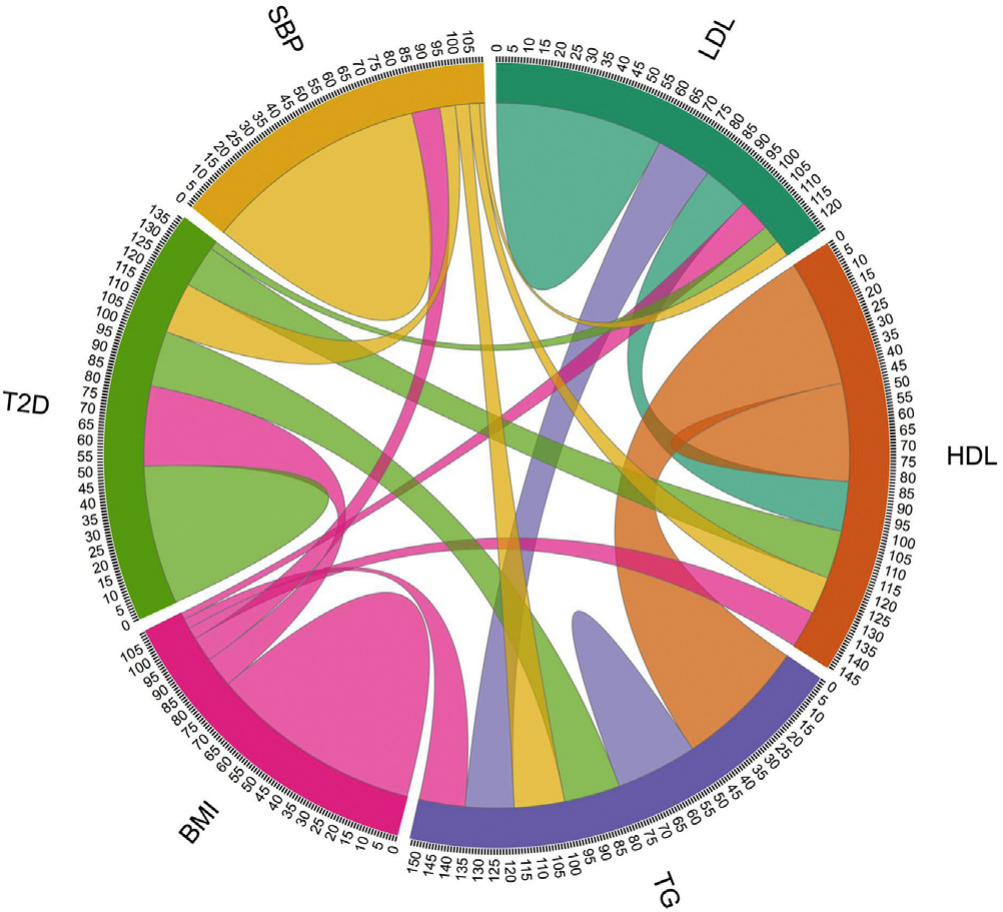
Pleiotropic effects of lipid trait SNPs on metabolic syndrome traits. In this chord plot, the thickness of the arc leaving each exposure trait represents the percentage of the genome-wide significant exposure trait SNPs that are associated with the pleiotropic trait receiving the arc at a Bonferroni-corrected significance level of 2 × 10^−5^. The size of the parabola that arcs from each exposure trait to itself represents the percentage of SNPs with no pleiotropic associations detected at a Bonferroni-corrected significance level of 2 × 10^−5^.

A total of 2,338 SNPs representing 1,492 distinct loci modifying lipid and metabolic syndrome traits at genome-wide significance were assessed for pleiotropy. Among all lipid trait SNPs, 30% have pleiotropic effects on BMI, T2D, or SBP at a Bonferroni-corrected significance threshold of *P* < 2 × 10^−5^, including 20% of LDL-C SNPs, 31% of HDL-C SNPs, and 37% of TG SNPs (Table 1; Fig. 1). Overlap between genetic determinants of HDL-C and TG is also widespread, with 47% of HDL-C SNPs affecting TG and 49% of TG SNPs affecting HDL-C (Table 1). As a result, only 36% of genome-wide significant HDL-C SNPs and 30% of genome-wide significant TG SNPs do not have effects on other lipid and metabolic syndrome traits (Fig. 1). A similar degree of genetic overlap between lipid and metabolic syndrome traits is evident in the set of 185 lipid trait SNPs based on the Global Lipid Genetics Consortium GWAS (Supplemental Table S1) that has been used in previous multivariable MR of lipid traits in CAD (8).

**Table 1:** Genetic overlap between lipid and metabolic syndrome traits

Exposure	Pleiotropic Trait						BMI,T2D, or SBP
	LDL-C	HDL-C	TG	BMI	T2D	SBP	
LDL-C	100%	17%	21%	12%	7%	6%	20%
HDL-C	18%	100%	47%	14%	18%	14%	31%
TG	18%	49%	100%	17%	21%	19%	37%
BMI	3%	4%	4%	100%	11%	9%	100%
T2D	3%	16%	21%	29%	100%	19%	100%
SBP	2%	4%	5%	10%	6%	100%	100%

Percent of SNPs that are genome-wide significant (*P* < 1 × 10^−8^) for the exposure trait with a secondary (pleiotropic trait) exposure that is significant after Bonferroni correction (*P* < 2 × 10^−5^).

Because many lipid trait SNPs carry pleiotropic lipid and metabolic syndrome trait effects, we first considered whether a restricted set of nonpleiotropic lipid trait SNPs could be generated by filtering out known pleiotropic SNPs. We generated prospective restricted sets of lipid trait-specific SNPs for HDL-C and TGs, where pleiotropy is most widespread, by filtering out SNPs with evidence for pleiotropy at a stringent Bonferroni-corrected threshold of *P* < 2 × 10^−5^. However, the distribution of *P*-values for pleiotropic trait effects in these restricted sets is concentrated in the interval *P* < 0.1 (Supplemental Fig. S2A, B), indicating that many of these variants have pleiotropic effects that are inefficiently estimated and not removed by filtering. Filtering out pleiotropic effects at *P* < 0.05 also yields a left-shifted distribution of *P*-values, suggesting that pleiotropic effects are still present (Supplemental Fig. S2C, D). Filtering out pleiotropic effects at *P* < 0.1 yields only six HDL-C and two TG SNPs (Supplemental Fig. S2E, F), an insufficient number for robust MR analysis. Thus, statistical filtering for nonpleiotropic lipid trait SNPs is limited by widespread pleiotropy, making multivariable adjustment a more appropriate approach for genome-wide MR of lipid traits in CAD.

To identify individual SNPs that modify CAD through lipid trait-independent mechanisms, we used the multivariable MR-PRESSO method (33) to test for large CAD effects that are not explained by effects on LDL-C, HDL-C, or TGs. Multivariable MR-PRESSO identified eight SNPs as pleiotropic in MR of lipid traits in CAD (Supplemental Table S2). Four of these have very large CAD effects and small lipid trait effects; these SNPs are in the *LPA* and 9p21 loci, which are well-known to have lipid trait-independent atherogenic effects (36, 37). Three of the remaining four lipid trait SNPs identified as pleiotropic by MR-PRESSO have strong SBP associations; the last SNP is located in *DAGLB*, the gene encoding diacylglycerol lipase β, in which a coding variant is associated with waist-hip ratio (38). In multivariable MR-PRESSO analysis of lipid and metabolic syndrome traits in CAD, only three SNPs in the *LPA* and 9p21 loci are identified by MR-PRESSO as pleiotropic (Supplemental Table S2). This indicates that their CAD effects are not mediated via the lipid and metabolic syndrome traits considered here but more likely through other mechanisms.

### Multivariable MR of lipid traits in CAD

**Fig. 2:**
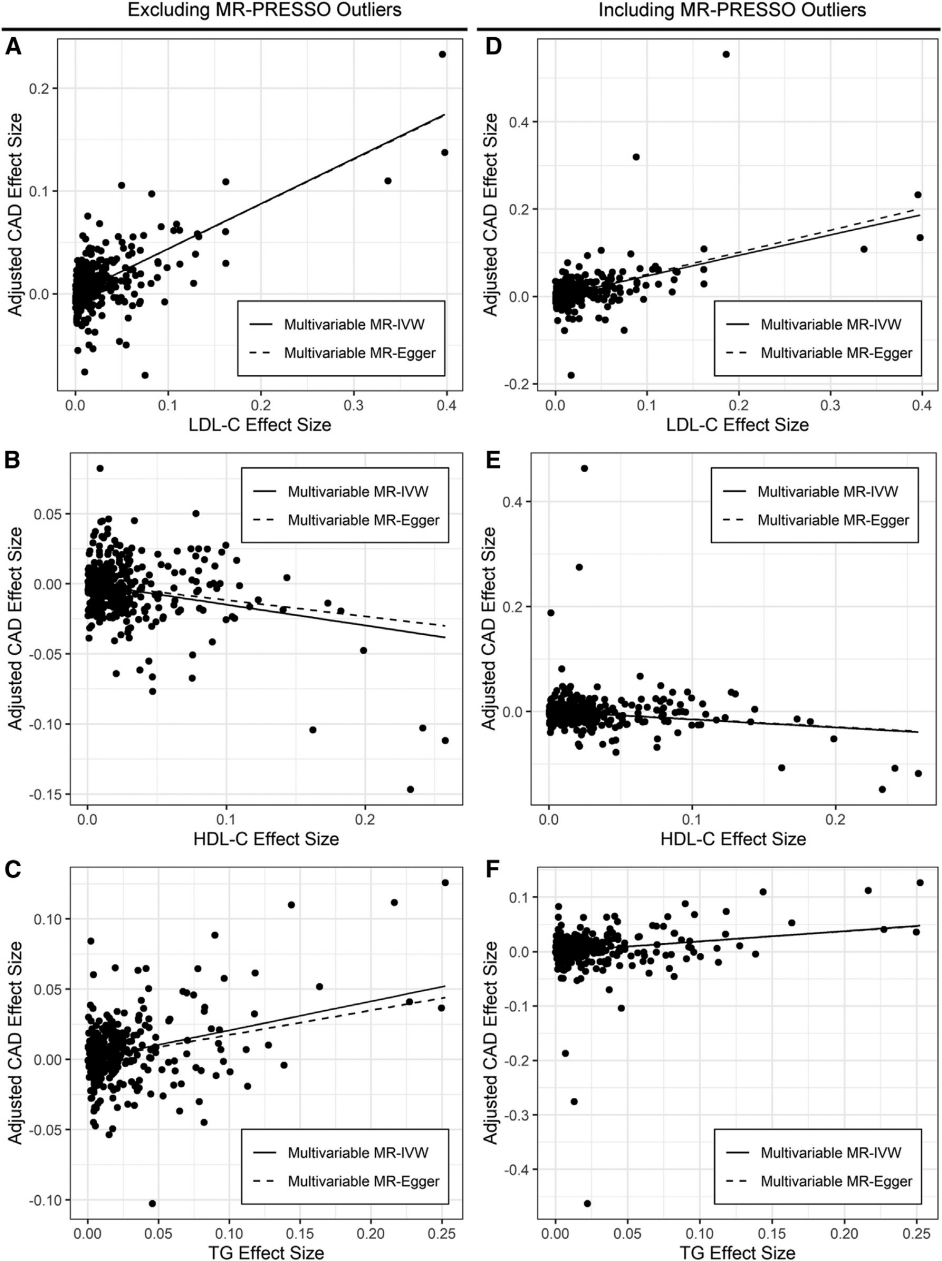
MR of lipid traits in CAD. Points represent genome-wide significant lipid trait SNPs without missing data. Regression lines for multivariable MR-IVW and multivariable MR-Egger are shown. A–C: Plot of LDL-C (A), HDL-C (B), or TG (C) effect size in SD units against lipid covariate-adjusted CAD risk in log OR, excluding eight pleiotropic SNPs identified by MR-PRESSO. D–F: Plot of LDL-C (D), HDL-C (E), or TG (F) effect size in SD units against lipid covariate-adjusted CAD risk in log OR, including eight pleiotropic SNPs identified by MR-PRESSO.

We used 343 genome-wide significant lipid trait SNPs to model multivariable MR of lipid traits in CAD using the Million Veteran Program lipid dataset and a recent meta-analysis of CARDIoGRAMplusC4D and UKBB datasets (n = 122,733 cases and 424,528 controls; 2-fold more CAD cases than CARDIoGRAMplusC4D alone) while excluding pleiotropic SNPs identified statistically by MR-PRESSO. The multivariable MR-IVW method accounts for effects of SNPs that modify multiple lipid traits. Multivariable MR-IVW analysis of lipid traits in CAD supports associations with CAD for LDL-C (OR 1.55; 95% CI 1.47–1.63; *P* = 4.8 × 10^−47^, HDL-C (OR 0.86; 95% CI 0.82–0.91; *P* = 1.2 × 10^−7^), and TGs (OR 1.23; 95% CI 1.16–1.30; *P* = 1.5 × 10^−11^) (Table 2; Fig. 2A–C).

**Table 2:** Multivariable MR of lipid traits in CAD

	Multivariable MR-IVW				Multivariable MR-Egger					
	OR	CI	*P*	I^2^	OR	CI	*P*	I^2^	Int.	Int. *P*
LDL-C	1.55	1.47–1.63	4.8 × 10^−47^	71%	1.55	1.46–1.65	5.0 × 10^−35^	71%	0.00011	0.92
HDL-C	0.86	0.82–0.91	1.2 × 10^−7^	71%	0.89	0.83–0.95	0.00089	71%	−0.0016	0.13
TG	1.23	1.16–1.30	1.5 × 10^−11^	71%	1.19	1.11–1.28	1.5 × 10^−6^	71%	0.0017	0.10

Results of multivariable regression of CAD effect size on LDL-C, HDL-C, and TG effect size with the intercept fixed at zero (multivariable MR-IVW) or with estimation of the intercept (multivariable MR-Egger). Eight pleiotropic SNPs identified by MR-PRESSO have been excluded; analysis with inclusion of these SNPs is presented in Supplemental Table S3. Int., MR-Egger intercept. OR, odds ratio per unit change in exposure trait.

Inclusion of eight pleiotropic SNPs identified by MR-PRESSO does not change this pattern of associations but decreases the strength of all associations slightly and increases the proportion of variation attributable to heterogeneity in the size of the lipid trait-CAD association for different SNPs (I^2^) by 19% (Supplemental Table S3; Fig. 2D–F). Multivariable MR-Egger estimates and adjusts for the intercept predicted by a collection of SNPs (11). The MR-Egger intercept can be interpreted as the CAD effect suggested by these SNPs in the absence of a lipid effect, e.g., a global measure of the unmeasured pleiotropic effects of a set of SNPs (4). Multivariable MR-Egger analysis moderately attenuates the HDL-C and TG associations (Table 2). However, the MR-Egger intercept test does not indicate the presence of bias due to directional pleiotropy.

### Multivariable MR of lipid and metabolic syndrome traits in CAD

**Fig. 3:**
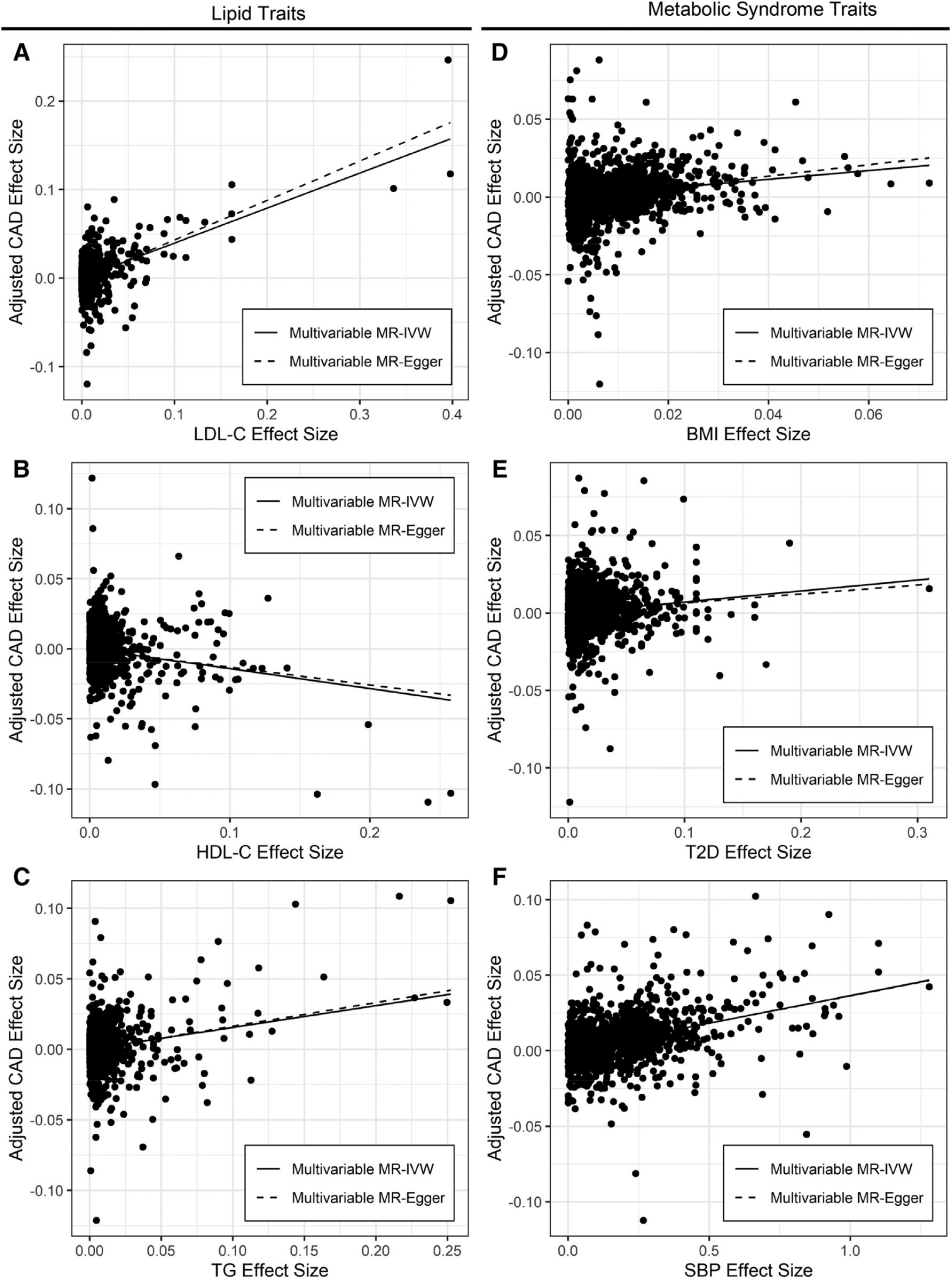
MR of lipid and metabolic syndrome traits in CAD. Points represent genome-wide significant lipid and metabolic syndrome trait SNPs without missing data. Regression lines for multivariable MR-IVW and multivariable MR-Egger are shown. A–F: Plot of LDL-C (A), HDL-C (B), TG (C), BMI (D), T2D (E), or SBP (F) effect size in SD units (LDL-C, HDL-C, TG, and BMI), log OR (T2D), or untransformed (SBP) against lipid and metabolic syndrome covariate-adjusted CAD risk in log OR, excluding three pleiotropic SNPs identified by MR-PRESSO.

Multivariable MR-IVW analysis of lipid and metabolic syndrome traits in CAD using 1,492 independent genome-wide significant lipid and metabolic syndrome trait SNPs reveals associations with CAD for LDL-C (OR 1.48; 95% CI 1.41–1.56; *P* = 4.5 × 10^−51^), HDL-C (OR 0.87; 95% CI 0.82–0.91; *P* = 1.1 × 10^−7^), TGs (OR 1.17; 95% CI 1.10–1.23; *P* = 4.8 × 10^−8^), BMI (OR 1.33; 95% CI 1.25–1.41; 2.2 × 10^−21^), T2D (OR 1.07; 95% CI 1.05–1.10; *P* = 3.3 × 10^−8^), and SBP (OR 1. 037; 95% CI 1.034–1.041; *P* = 3.5 × 10^−91^) when the three pleiotropic SNPs identified by MR-PRESSO are excluded (Table 3; Fig. 3A–F). Inclusion of three pleiotropic SNPs identified by MR-PRESSO does not change this pattern but decreases the strength of all associations slightly and increases the proportion of variation attributable to heterogeneity in the size of the lipid trait-CAD association for different SNPs (I^2^) by 7% (Supplemental Table S4).

**Table 3:** Multivariable MR of lipid and metabolic syndrome traits in CAD

	Multivariable MR-IVW				Multivariable MR-Egger					
	OR	CI	*P*	I^2^	OR	CI	*P*	I^2^	Int.	Int. *P*
LDL-C	1.48	1.41–1.56	4.5 × 10^−51^	70%	1.56	1.48–1.65	3.8 × 10^−54^	70%	−0.0015	1.0 × 10^−5^
HDL-C	0.87	0.82–0.91	1.1 × 10^−7^	70%	0.88	0.83–0.93	1.4 × 10^−5^	70%	−0.00053	0.19
TG	1.17	1.10–1.23	4.8 × 10^−8^	70%	1.18	1.11–1.26	6.2 × 10^−8^	70%	−0.00041	0.30
BMI	1.33	1.25–1.41	2.2 × 10^−21^	70%	1.44	1.32–1.57	3.3 × 10^−16^	70%	−0.0012	0.013
T2D	1.07	1.05–1.10	3.3 × 10^−8^	70%	1.06	1.03–1.09	0.00020	70%	0.00071	0.13
SBP	1.037	1.034–1.041	3.5 × 10^−91^	70%	1.037	1.032–1.042	1.3 × 10^−50^	70%	7.7 × 10^−5^	0.86

Results of multivariable regression of CAD effect size on LDL-C, HDL-C, TG, BMI, T2D, and SBP effect size with the intercept fixed at zero (multivariable MR-IVW) or with estimation of the intercept (multivariable MR-Egger). Three pleiotropic SNPs identified by MR-PRESSO have been excluded; analysis with inclusion of these SNPs is presented in Supplemental Table S4. Int., MR-Egger intercept; OR, odds ratio per unit change in exposure trait.

Multivariable MR-Egger analysis of lipid and metabolic syndrome traits in CAD also suggests associations of LDL-C, HDL-C, TGs, BMI, T2D, and SBP with CAD. The MR-Egger intercept test does not indicate the presence of bias due to directional pleiotropy for HDL-C, TGs, T2D, and SBP. However, it does indicate the presence of bias due to directional pleiotropy for LDL-C and BMI, and MR-Egger adjustment moderately increases the OR for LDL-C and BMI. This effect suggests that the LDL-C and BMI estimates are affected by pleiotropic effects of the modeled SNPs that are still unaccounted for, and that the magnitude of these associations may be moderately larger if these factors were modeled.

### Monogenic univariable MR of lipid traits in CAD

In multivariable MR of lipid and metabolic syndrome traits, a moderate to high degree of statistical heterogeneity remains (I^2^ = 70%), likely reflecting distinct pathways by which different SNPs modify lipid and metabolic syndrome traits and consequent variation in the respective CAD associations (Table 3). To characterize this heterogeneity, we performed monogenic univariable MR at lipid trait-modifying loci where the mechanism of the lipid trait effect is well-defined, facilitating the causal interpretation of monogenic MR at these loci (39, 40). We focused on HDL-C and TGs for this analysis because the causal effect of LDL-C in CAD is well-established and the effects of LDL-C SNPs on CAD are relatively homogeneous (Fig. 2A); monogenic univariable MR of mechanistically well-defined LDL-C SNPs of large lipid trait effect size, e.g., *PCSK9* and *LDLR* (Supplemental Table S5), has been previously reported (41).

We first considered all loci carrying HDL-C and TG SNPs with large lipid trait effect sizes, which are listed in Supplemental Table S5. Of these loci, *LIPG*, *CETP*, *LPL*, and *ANGTPL4* have well-defined mechanisms linked to a single enzymatic activity, while SNPs at the *CD300LG* and *APOA1-C3* loci do not. Variants in *CETP* were previously studied and have been suggested to modify CAD risk via disparate effects on multiple lipid traits (42, 43, 44, 45), so we omitted this locus from monogenic univariable MR. In order to interrogate the basis of heterogeneity of HDL-C and TG associations with CAD, we also selected mechanistically well-defined loci carrying SNPs with moderate lipid trait effects and CAD effects that are inconsistent with the CAD effects of large effect SNPs, which occurred at *LCAT* and *LIPC* for HDL-C, and *MLXIPL* and *FADS1* for TG.

### Locus-specific MR at HDL-C-modifying genes

**Fig. 4:**
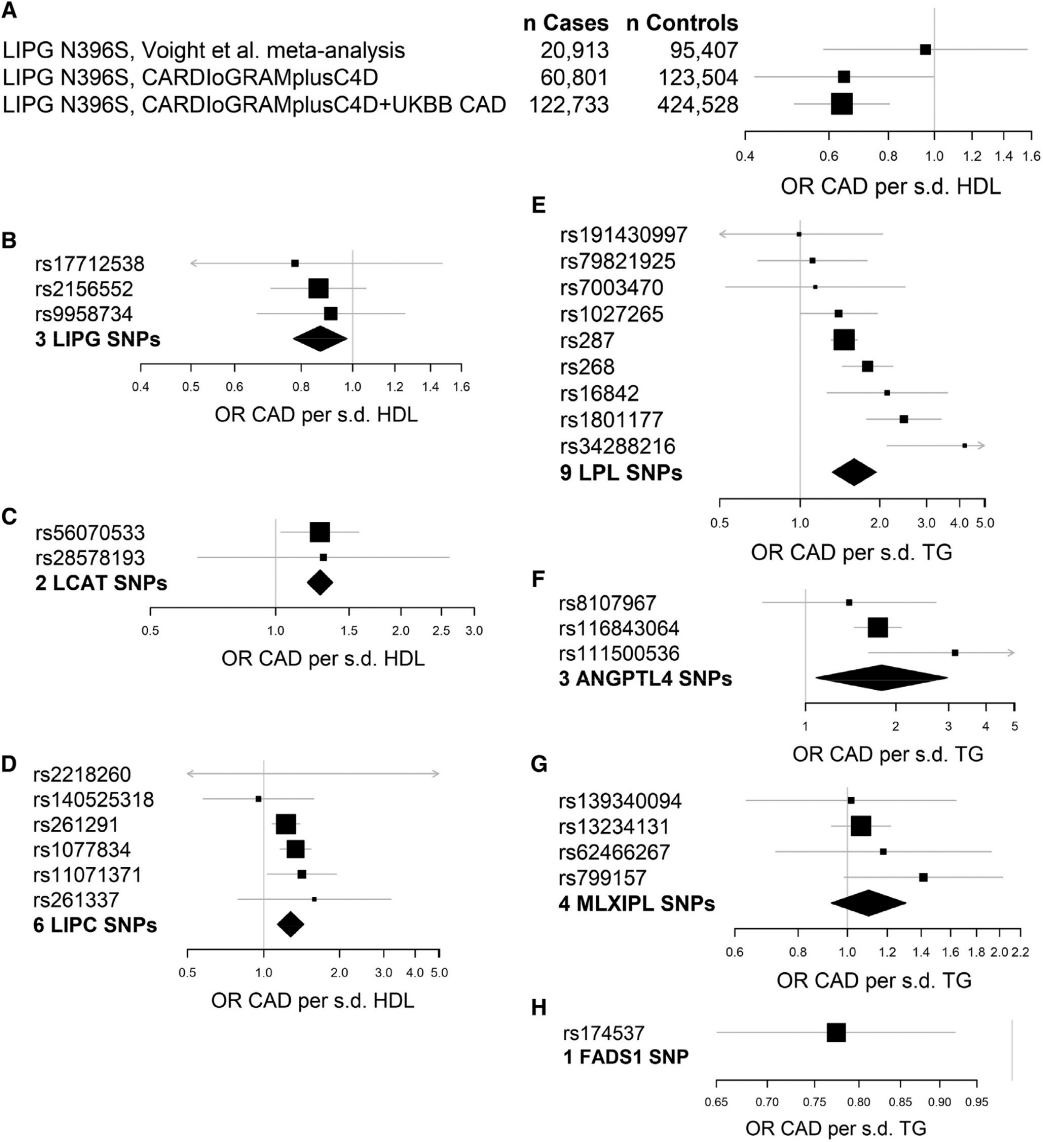
Locus- and mechanism-specific MR associations for HDL-C and TG. Forest plots of locus- or SNP-specific MR estimates for the indicated genes. A: MR of HDL-C in CAD based on the LIPG N396S mutation in successive CAD datasets of increasing size. B: MR of HDL-C in CAD based on three noncoding SNPs in the *LIPG* locus. C: MR of HDL-C in CAD based on two SNPs in the *LCAT* locus. D: MR of HDL-C in CAD based on six SNPs in the *LIPC* locus. E: MR of TG in CAD based on nine SNPs in the *LPL* locus. F: MR of TGs in CAD based on three SNPs in the *ANGPTL4* locus. G: MR of TGs in CAD based on four SNPs in the *MLXIPL* locus. H: MR of TGs in CAD based on one SNP in the *FADS1* locus.

We performed monogenic univariable MR of HDL-C in CAD at *LIPG*, *LCAT*, and *LIPC*. The *LIPG* locus encodes endothelial lipase, which modifies HDL-C by catabolizing HDL phospholipids; this activity decreases HDL stability and may cause the resulting phospholipid-poor HDL to be a poorer cholesterol acceptor (46, 47, 48). The rs77960347 SNP in *LIPG* encoding the N396S mutation confers a large increase in HDL-C and has been previously studied in a monogenic MR experiment (6). In MR based on CAD data from 20,913 CAD cases and 95,407 controls, a null association of LIPG N396S with CAD was reported (6), with an OR for CAD of 0.99 (95% CI 0.88–1.11; *P* = 0.85) for the G allele encoding the missense mutation. In a subsequent CAD GWAS comprising 60,801 CAD cases and 123,504 controls (49), LIPG N396S is associated with a possible CAD effect with an OR of 0.90 (95% CI 0.82–1.00; *P* = 0.05) per G allele. In the present CAD meta-analysis of 122,733 CAD cases and 424,528 controls, LIPG N396S is associated with a CAD effect with an OR of 0.90 (95% CI 0.86–0.95; *P* = 8.8 × 10^−5^) per G allele. Consequently, univariable MR on this single SNP reveals a causal protective effect of increased HDL-C conferred by decreased endothelial lipase activity in the present study (Table 4; Fig. 4A, shown as OR per SD increase in HDL-C).

**Table 4:** Monogenic univariable MR of HDL-C and TG in CAD

Locus	Trait	Univariable MR-IVW			
		OR	CI	*P*	I^2^
LIPG N396S^a^	HDL-C	0.64	0.51–0.80	0.00013	—
3 Regulatory *LIPG* SNPs	HDL-C	0.87	0.77–0.98	0.035	0%
2 *LCAT* SNPs	HDL-C	1.28	1.19–1.37	0.014	0%
6 *LIPC* SNPs	HDL-C	1.28	1.13–1.44	0.0032	8%
9 *LPL* SNPs	TG	1.60	1.33–1.93	0.00043	72%
3 *ANGPTL4* SNPs	TG	1.79	1.08–2.97	0.038	50%
4 *MLXIPL* SNPs	TG	1.10	0.93–1.31	0.16	0%
1 *FADS1* SNP	TG	0.77	0.65–0.92	0.0038	—

Results of univariable MR on selected SNPs at seven representative loci for genes directly modifying HDL-C or TG. The heterogeneity statistic I^2^ is reported for analyses involving more than one SNP. OR, odds ratio per unit change in HDL-C or TG.

a Based on CAD data from the meta-analysis of CARDIoGRAMplusC4D and UKBB data.

Three SNPs in regulatory regions of the *LIPG* locus, including the UTR and upstream and downstream noncoding DNA, are associated with smaller HDL-C effects and concordant small CAD effects. In univariable MR on these three regulatory variants in *LIPG*, increased HDL-C is associated with decreased CAD risk (Table 4; Fig. 4B). Mutations in *LIPG* have effects on LDL-C at 25–35% the magnitude of their HDL-C effects, but these LDL-C effects are directionally discordant with the CAD effects of *LIPG* SNPs, e.g., inconsistent with mediation via a deleterious effect of LDL-C (Supplemental Table S6).

The *LCAT* locus encodes LCAT, which modifies HDL-C by converting free cholesterol in HDL into the more neutral species cholesteryl ester without affecting macrophage reverse cholesterol transport (50). SNPs in *LCAT* lead to isolated changes in HDL-C (Supplemental Table S6) and confer an increased risk of CAD per unit increase in HDL-C (Table 4; Fig. 4C). The *LIPC* locus encodes hepatic lipase, which remodels HDL and apoB-containing lipoproteins (51) and modifies HDL-C by enhancing hepatic selective uptake of cholesteryl esters without affecting macrophage reverse cholesterol transport (52, 53, 54). SNPs in *LIPC* primarily modify HDL-C, except for the LDL-C SNP rs2218260 (Supplemental Table S6). SNPs in *LIPC* confer an increased risk of CAD per unit increase in HDL-C; this effect is independent of rs2218260 (Table 4; Fig. 4D).

### Locus-specific MR at TG-modifying genes

We next performed monogenic univariable MR of TGs in CAD at *LPL*, *ANGTPL4*, *MLXIPL*, and *FADS1*. Nine distinct SNPs in *LPL* encoding LPL, which hydrolyzes TGs in all lipoprotein classes (55), are associated with large and heterogeneous, but consistently deleterious, effects on CAD per unit increase in TGs. Univariable MR on this set of nine SNPs suggests that increased TGs conferred by decreased LPL activity leads to increased CAD risk (Table 4; Fig. 4E), as previously reported (7, 56). SNPs at *ANGPTL4* encoding angiopoietin-like 4, an inhibitor of LPL (57), are also associated with large deleterious effects on CAD per unit increase in TGs. Univariable MR on three *ANGPTL4* SNPs suggests that increased TGs conferred by *ANGPTL4*-mediated LPL inhibition leads to increased CAD risk (Table 4; Fig. 4F), consistent with prior studies (7, 58). SNPs in both *LPL* and *ANGPTL4* are associated with large HDL-C effects in addition to their TG effects (Supplemental Tables S5, S7), likely as a consequence of increased cholesteryl ester transfer protein activity driven by availability of TGs (59).

We also considered TG SNPs at the transcriptional regulator *MLXIPL* (also known as carbohydrate response element binding protein), which controls expression of genes involved in hepatic TG biosynthesis (60). When considered in univariable MR, four SNPs in *MLXIPL* were not associated with an increased risk of CAD per unit increase in TGs (Table 4; Fig. 4G). The *FADS1* locus, which encodes a fatty acid desaturase that produces both substrates and endogenous regulators of TG biosynthesis (61), is represented by one distinct SNP, rs174537, which primarily modifies TGs. In univariable MR, rs174537 is associated with decreased risk of CAD per unit increase in TGs (Table 4; Fig. 4H). SNPs in *MLXIPL* and *FADS1* have HDL-C effects at 25–50% of the magnitude of the TG effects that do not account for the lack of deleterious TG effects observed at these loci (Supplemental Table S7).

## Discussion

In this study, data from large GWASs of lipid and metabolic traits reveal that numerous lipid trait SNPs also have effects on metabolic syndrome traits. These widespread pleiotropic effects invalidate the use of polygenic univariable MR using restricted sets of SNPs apparently specific for a single lipid trait, even though this approach was used in early MR studies of lipid traits in CAD (6, 34, 62). When pleiotropic effects are accounted for in multivariable MR, we show independent associations between LDL-C, HDL-C, TGs, BMI, T2D, and SBP with CAD, and each of these associations is robust to adjustment for directional pleiotropy using the MR-Egger method. Our results suggest that the lipid and metabolic syndrome traits LDL-C, HDL-C, TGs, BMI, T2D and SBP are causally linked to CAD, although HDL-C and TGs appear to have locus- and mechanism-specific causal effects.

The CAD associations for LDL-C, TGs, BMI, T2D, and SBP are consistent with the results of previous MR and observational studies (9, 63, 64, 65, 66, 67, 68, 69). In previous smaller studies, MR of lipid traits in CAD revealed causal associations of LDL-C and TGs with CAD and suggested that pleiotropic effects of lipid trait SNPs accounted for much of the apparent protective effect of HDL-C and part of the deleterious effect of TG (9, 18, 19). Here, we have systematically addressed pleiotropy due to co-occurrence of lipid and metabolic trait derangements in insulin resistance and the metabolic syndrome using multivariable analysis on large GWAS datasets, with the result that there is minimal evidence of effects of residual pleiotropy that would substantially alter our conclusions. Our finding that LDL-C, HDL-C, and TGs are independently associated with CAD by MR builds on three recent reports showing independent MR associations of LDL-C, HDL-C, and TGs with abdominal aortic aneurysm (19, 20, 70), suggesting that each of these factors is biologically active at the level of the arterial wall.

**Fig. 5:**
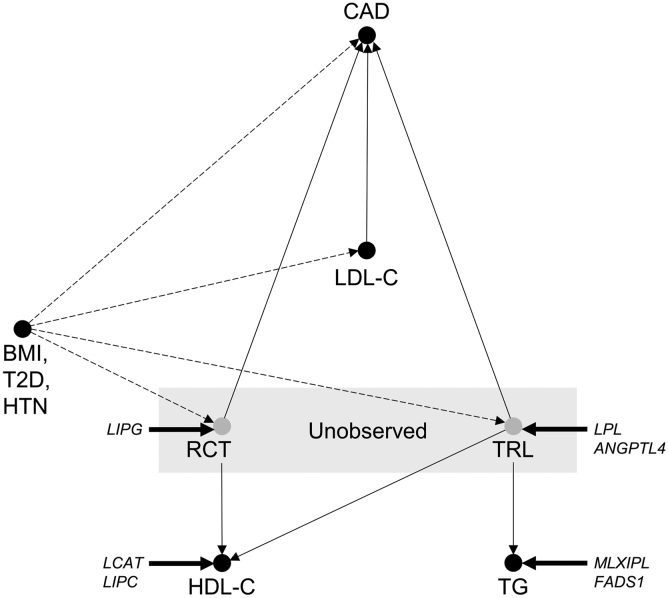
Proposed causal diagram of lipid and metabolic syndrome trait effects in CAD. Directed acyclic graph representing proposed causal relationships between lipid traits and CAD, unobserved causal factors mediating HDL-C and TG effects, and confounding by metabolic syndrome traits. Putative effect of genetic intervention at *LIPG, LCAT, LIPC, LPL, ANGPTL4, MLXIPL*, and *FADS1* is indicated.

The locus- and mechanism-specific causal effects of HDL-C and TGs may be expected given the biology of the lipid traits. While LDL-C is thought to directly measure the primary etiologic agent of CAD (71), the HDL hypothesis suggests that HDL-C is a proxy for an unmeasured causal variable, macrophage cholesterol efflux and reverse cholesterol transport, which mediate protection from atherosclerosis (Fig. 5) (72, 73). Increased HDL-C conferred by decreased endothelial lipase activity is linked to protection from CAD, possibly because the change in HDL-C in this setting reflects a change in properties of HDL that correlate with increased cholesterol efflux and reverse cholesterol transport (47, 48). In contrast, mutations in LCAT and LIPC that increase the cholesteryl ester content of HDL without concordantly modifying reverse cholesterol transport (50, 53) lead to increased HDL-C but are associated with increased CAD.

Likewise, TG is a proxy for the concentration and composition of TG-rich lipoproteins (TRLs), which promote atherosclerosis via several mechanisms, including deposition of cholesterol and apoB in atherosclerotic lesions (Fig. 5) (74, 75). SNPs in *LPL* and *ANGTPL4* that increase TG by decreasing the turnover of TRL have large deleterious effects on CAD, but SNPs in *MLXIPL* and *FADS1* that modify TG biosynthesis have minimal or opposite effects on CAD. Our conclusion that HDL-C and TGs are associated with locus- and mechanism-specific causal effects on CAD is not in disagreement with the negative results of some randomized controlled trials of HDL-C-raising and TG-lowering therapies, including fibrates, niacin, and cholesteryl ester transfer protein inhibitors (76, 77, 78, 79), as the reported MR associations represent a composite of hundreds of distinct lipid-modifying mechanisms (17).

Certain characteristics of the datasets used in this study are unique. Using a densely imputed GWAS of 300,000 individuals, we identified twice as many lipid trait SNPs compared with previous lipid trait MR and estimated their CAD effects from a dataset over twice as large as the CAD datasets used in previous lipid trait MR (8, 9, 18, 19). Metabolic syndrome trait effects for lipid and metabolic syndrome trait SNPs were estimated from GWASs of >700,000 individuals, and we used this data to adjust for metabolic syndrome trait effects and reduce confounding due to pleiotropic effects of lipid trait SNPs. Using MR-PRESSO, we screened for SNPs with large exposure-independent CAD effects, including SNPs in the *LPA* and 9p21 loci. Exclusion of these SNPs reduced the potential for bias due to direct CAD effects of lipid trait SNPs, decreased statistical heterogeneity, and permitted more robust multivariable MR of lipid and metabolic syndrome traits.

Our study also has several limitations. We adjusted for pleiotropic effects of lipid SNPs at the level of metabolic syndrome traits and pathways leading to large inconsistent CAD effects, but the moderate to high degree of heterogeneity in our final multivariable MR model suggests that genetic overlap with unmeasured traits is present. MR-Egger analysis suggests that pleiotropic effects of these SNPs do not bias the modeled associations for HDL-C, TGs, T2D, and SBP, while adjustment for directional pleiotropy slightly increases the magnitude of the LDL-C and BMI associations and does not change our conclusions. We used large datasets to model lipid and metabolic syndrome traits and CAD, but the quality of effect size estimates is likely to improve with the incorporation of additional data. This is particularly evident for CAD, as illustrated by the improvement in precision over successive CAD datasets for the LIPG N396S mutation. Finally, while MR may provide insights into causal effects of the exposure traits, it may not accurately estimate the magnitude of those effects (2, 17, 40) and, thus, may have limited utility in assessing the impact of therapeutic intervention on the clinical outcome.

### Data availability

All GWAS summary statistics used for the included MR analyses and analysis of pleiotropy have been deposited and published on Mendeley: https://data.mendeley.com/datasets/dz9w684x8p/1 (https://doi.org/10.17632/dz9w684x8p.1). R scripts for these analyses are available on Github: https://www.github.com/dgt2109/bio-script/ (https://doi.org/10.5281/zenodo.3986183).

## Conflict of interest

A.R.T. is a consultant to CSL and Amgen and on the scientific advisory boards of Staten Biotechnology and Fortico Biotechnology.

## References

[1] Lawlor D.A., Harbord R.M., Sterne J.A., Timpson N., Davey Smith G. (2008). Mendelian randomization: using genes as instruments for making causal inferences in epidemiology. Stat. Med..

[2] Burgess S., Butterworth A., Malarstig A., Thompson S.G. (2012). Use of Mendelian randomisation to assess potential benefit of clinical intervention. BMJ.

[3] Palmer T.M., Lawlor D.A., Harbord R.M., Sheehan N.A., Tobias J.H., Timpson N.J., Davey Smith G., Sterne J.A. (2012). Using multiple genetic variants as instrumental variables for modifiable risk factors. Stat. Methods Med. Res..

[4] Bowden J., Davey Smith G., Burgess S. (2015). Mendelian randomization with invalid instruments: effect estimation and bias detection through Egger regression. Int. J. Epidemiol..

[5] Linsel-Nitschke P., Gotz A., Erdmann J., Braenne I., Braund P., Hengstenberg C., Stark K., Fischer M., Schreiber S., El Mokhtari N.E. (2008). Lifelong reduction of LDL-cholesterol related to a common variant in the LDL-receptor gene decreases the risk of coronary artery disease–a Mendelian Randomisation study. PLoS One.

[6] Voight B.F., Peloso G.M., Orho-Melander M., Frikke-Schmidt R., Barbalic M., Jensen M.K., Hindy G., Holm H., Ding E.L., Johnson T. (2012). Plasma HDL cholesterol and risk of myocardial infarction: a mendelian randomisation study. Lancet.

[7] Lotta L.A., Stewart I.D., Sharp S.J., Day F.R., Burgess S., Luan J., Bowker N., Cai L., Li C., Wittemans L.B.L. (2018). Association of genetically enhanced lipoprotein lipase-mediated lipolysis and low-density lipoprotein cholesterol-lowering alleles with risk of coronary disease and type 2 diabetes. JAMA Cardiol.

[8] Do R., Willer C.J., Schmidt E.M., Sengupta S., Gao C., Peloso G.M., Gustafsson S., Kanoni S., Ganna A., Chen J. (2013). Common variants associated with plasma triglycerides and risk for coronary artery disease. Nat. Genet..

[9] White J., Swerdlow D.I., Preiss D., Fairhurst-Hunter Z., Keating B.J., Asselbergs F.W., Sattar N., Humphries S.E., Hingorani A.D., Holmes M.V. (2016). Association of lipid fractions with risks for coronary artery disease and diabetes. JAMA Cardiol.

[10] Burgess S., Thompson S.G. (2015). Multivariable Mendelian randomization: the use of pleiotropic genetic variants to estimate causal effects. Am. J. Epidemiol..

[11] Rees J.M.B., Wood A.M., Burgess S. (2017). Extending the MR-Egger method for multivariable Mendelian randomization to correct for both measured and unmeasured pleiotropy. Stat. Med..

[12] Bulik-Sullivan B., Finucane H.K., Anttila V., Gusev A., Day F.R., Loh P.R., ReproGen Consortium, Psychiatric Genomics Consortium, Genetic Consortium for Anorexia Nervosa of the Wellcome Trust Case Control Consortium 3, Duncan L. (2015). An atlas of genetic correlations across human diseases and traits. Nat. Genet..

[13] Pickrell J.K., Berisa T., Liu J.Z., Segurel L., Tung J.Y., Hinds D.A. (2016). Detection and interpretation of shared genetic influences on 42 human traits. Nat. Genet..

[14] Emdin C.A., Khera A.V., Natarajan P., Klarin D., Zekavat S.M., Hsiao A.J., Kathiresan S. (2017). Genetic association of waist-to-hip ratio with cardiometabolic traits, type 2 diabetes, and coronary heart disease. JAMA.

[15] Shu L., Chan K.H.K., Zhang G., Huan T., Kurt Z., Zhao Y., Codoni V., Tregouet D.A., Cardiogenics C., Yang J. (2017). Shared genetic regulatory networks for cardiovascular disease and type 2 diabetes in multiple populations of diverse ethnicities in the United States. PLoS Genet.

[16] Grundy S.M., Cleeman J.I., Daniels S.R., Donato K.A., Eckel R.H., Franklin B.A., Gordon D.J., Krauss R.M., Savage P.J., Smith S.C. (2005). Diagnosis and management of the metabolic syndrome: an American Heart Association/National Heart, Lung, and Blood Institute Scientific Statement. Circulation.

[17] Burgess S., Freitag D.F., Khan H., Gorman D.N., Thompson S.G. (2014). Using multivariable Mendelian randomization to disentangle the causal effects of lipid fractions. PLoS One.

[18] Zhu Z., Zheng Z., Zhang F., Wu Y., Trzaskowski M., Maier R., Robinson M.R., McGrath J.J., Visscher P.M., Wray N.R. (2018). Causal associations between risk factors and common diseases inferred from GWAS summary data. Nat. Commun..

[19] Allara E., Morani G., Carter P., Gkatzionis A., Zuber V., Foley C.N., Rees J.M.B., Mason A.M., Bell S., Gill D. (2019). Genetic determinants of lipids and cardiovascular disease outcomes: a wide-angled Mendelian randomization investigation. Circ. Genom. Precis. Med..

[20] Klarin D., Damrauer S.M., Cho K., Sun Y.V., Teslovich T.M., Honerlaw J., Gagnon D.R., DuVall S.L., Li J., Peloso G.M. (2018). Genetics of blood lipids among ∼300,000 multi-ethnic participants of the Million Veteran Program. Nat. Genet..

[21] Pulit S.L., Stoneman C., Morris A.P., Wood A.R., Glastonbury C.A., Tyrrell J., Yengo L., Ferreira T., Marouli E., Ji Y. (2019). Meta-analysis of genome-wide association studies for body fat distribution in 694 649 individuals of European ancestry. Hum. Mol. Genet..

[22] Mahajan A., Taliun D., Thurner M., Robertson N.R., Torres J.M., Rayner N.W., Payne A.J., Steinthorsdottir V., Scott R.A., Grarup N. (2018). Fine-mapping type 2 diabetes loci to single-variant resolution using high-density imputation and islet-specific epigenome maps. Nat. Genet..

[23] Evangelou E., Warren H.R., Mosen-Ansorena D., Mifsud B., Pazoki R., Gao H., Ntritsos G., Dimou N., Cabrera C.P., Karaman I. (2018). Genetic analysis of over 1 million people identifies 535 new loci associated with blood pressure traits. Nat. Genet..

[24] van der Harst P., Verweij N. (2018). Identification of 64 novel genetic loci provides an expanded view on the genetic architecture of coronary artery disease. Circ. Res..

[25] Wu Y., Zheng Z., Visscher P.M., Yang J. (2017). Quantifying the mapping precision of genome-wide association studies using whole-genome sequencing data. Genome Biol.

[26] Kanai M., Tanaka T., Okada Y. (2016). Empirical estimation of genome-wide significance thresholds based on the 1000 Genomes Project data set. J. Hum. Genet..

[27] Fadista J., Manning A.K., Florez J.C., Groop L. (2016). The (in)famous GWAS P-value threshold revisited and updated for low-frequency variants. Eur. J. Hum. Genet..

[28] Hemani G., Zheng J., Elsworth B., Wade K.H., Haberland V., Baird D., Laurin C., Burgess S., Bowden J., Langdon R. (2018). The MR-Base platform supports systematic causal inference across the human phenome. eLife.

[29] Burgess S., Butterworth A., Thompson S.G. (2013). Mendelian randomization analysis with multiple genetic variants using summarized data. Genet. Epidemiol..

[30] Thomas D.C., Lawlor D.A., Thompson J.R. (2007). Re: Estimation of bias in nongenetic observational studies using “Mendelian triangulation” by Bautista et al. Ann. Epidemiol.

[31] Jackson D., White I.R., Riley R.D. (2012). Quantifying the impact of between-study heterogeneity in multivariate meta-analyses. Stat. Med..

[32] Higgins J.P., Thompson S.G., Deeks J.J., Altman D.G. (2003). Measuring inconsistency in meta-analyses. BMJ.

[33] Verbanck M., Chen C.Y., Neale B., Do R. (2018). Detection of widespread horizontal pleiotropy in causal relationships inferred from Mendelian randomization between complex traits and diseases. Nat. Genet..

[34] Willer C.J., Schmidt E.M., Sengupta S., Peloso G.M., Gustafsson S., Kanoni S., Ganna A., Chen J., Buchkovich M.L., Mora S. (2013). Discovery and refinement of loci associated with lipid levels. Nat. Genet..

[35] Lohmueller K.E., Pearce C.L., Pike M., Lander E.S., Hirschhorn J.N. (2003). Meta-analysis of genetic association studies supports a contribution of common variants to susceptibility to common disease. Nat. Genet..

[36] Clarke R., Peden J.F., Hopewell J.C., Kyriakou T., Goel A., Heath S.C., Parish S., Barlera S., Franzosi M.G., Rust S. (2009). Genetic variants associated with Lp(a) lipoprotein level and coronary disease. N. Engl. J. Med..

[37] McPherson R., Pertsemlidis A., Kavaslar N., Stewart A., Roberts R., Cox D.R., Hinds D.A., Pennacchio L.A., Tybjaerg-Hansen A., Folsom A.R. (2007). A common allele on chromosome 9 associated with coronary heart disease. Science.

[38] Justice A.E., Karaderi T., Highland H.M., Young K.L., Graff M., Lu Y., Turcot V., Auer P.L., Fine R.S., Guo X. (2019). Protein-coding variants implicate novel genes related to lipid homeostasis contributing to body-fat distribution. Nat. Genet..

[39] Burgess S., Foley C.N., Zuber V. (2018). Inferring causal relationships between risk factors and outcomes from genome-wide association study data. Annu. Rev. Genomics Hum. Genet..

[40] Burgess S., Butterworth A.S., Thompson J.R. (2016). Beyond Mendelian randomization: how to interpret evidence of shared genetic predictors. J. Clin. Epidemiol..

[41] Ference B.A., Robinson J.G., Brook R.D., Catapano A.L., Chapman M.J., Neff D.R., Voros S., Giugliano R.P., Davey Smith G., Fazio S. (2016). Variation in PCSK9 and HMGCR and risk of cardiovascular disease and diabetes. N. Engl. J. Med..

[42] Thompson A., Di Angelantonio E., Sarwar N., Erqou S., Saleheen D., Dullaart R.P., Keavney B., Ye Z., Danesh J. (2008). Association of cholesteryl ester transfer protein genotypes with CETP mass and activity, lipid levels, and coronary risk. JAMA.

[43] Nomura A., Won H.H., Khera A.V., Takeuchi F., Ito K., McCarthy S., Emdin C.A., Klarin D., Natarajan P., Zekavat S.M. (2017). Protein-truncating variants at the cholesteryl ester transfer protein gene and risk for coronary heart disease. Circ. Res..

[44] Ference B.A., Kastelein J.J.P., Ginsberg H.N., Chapman M.J., Nicholls S.J., Ray K.K., Packard C.J., Laufs U., Brook R.D., Oliver-Williams C. (2017). Association of genetic variants related to CETP inhibitors and statins with lipoprotein levels and cardiovascular risk. JAMA.

[45] Blauw L.L., Noordam R., Soidinsalo S., Blauw C.A., Li-Gao R., de Mutsert R., Berbee J.F.P., Wang Y., van Heemst D., Rosendaal F.R. (2019). Mendelian randomization reveals unexpected effects of CETP on the lipoprotein profile. Eur. J. Hum. Genet..

[46] Maugeais C., Tietge U.J., Broedl U.C., Marchadier D., Cain W., McCoy M.G., Lund-Katz S., Glick J.M., Rader D.J. (2003). Dose-dependent acceleration of high-density lipoprotein catabolism by endothelial lipase. Circulation.

[47] Yasuda T., Ishida T., Rader D.J. (2010). Update on the role of endothelial lipase in high-density lipoprotein metabolism, reverse cholesterol transport, and atherosclerosis. Circ. J..

[48] Agarwala A.P., Rodrigues A., Risman M., McCoy M., Trindade K., Qu L., Cuchel M., Billheimer J., Rader D.J. (2015). High-density lipoprotein (hdl) phospholipid content and cholesterol efflux capacity are reduced in patients with very high HDL cholesterol and coronary disease. Arterioscler. Thromb. Vasc. Biol..

[49] Nikpay M., Goel A., Won H.H., Hall L.M., Willenborg C., Kanoni S., Saleheen D., Kyriakou T., Nelson C.P., Hopewell J.C. (2015). A comprehensive 1,000 Genomes-based genome-wide association meta-analysis of coronary artery disease. Nat. Genet..

[50] Tanigawa H., Billheimer J.T., Tohyama J., Fuki I.V., Ng D.S., Rothblat G.H., Rader D.J. (2009). Lecithin:cholesterol acyltransferase expression has minimal effects on macrophage reverse cholesterol transport in vivo. Circulation.

[51] Santamarina-Fojo S., Gonzalez-Navarro H., Freeman L., Wagner E., Nong Z. (2004). Hepatic lipase, lipoprotein metabolism, and atherogenesis. Arterioscler. Thromb. Vasc. Biol..

[52] Collet X., Tall A.R., Serajuddin H., Guendouzi K., Royer L., Oliveira H., Barbaras R., Jiang X.C., Francone O.L. (1999). Remodeling of HDL by CETP in vivo and by CETP and hepatic lipase in vitro results in enhanced uptake of HDL CE by cells expressing scavenger receptor B-I. J. Lipid Res..

[53] Lambert G., Amar M.J., Martin P., Fruchart-Najib J., Foger B., Shamburek R.D., Brewer H.B., Santamarina-Fojo S. (2000). Hepatic lipase deficiency decreases the selective uptake of HDL-cholesteryl esters in vivo. J. Lipid Res..

[54] Ruel I.L., Couture P., Cohn J.S., Bensadoun A., Marcil M., Lamarche B. (2004). Evidence that hepatic lipase deficiency in humans is not associated with proatherogenic changes in HDL composition and metabolism. J. Lipid Res..

[55] Otarod J.K., Goldberg I.J. (2004). Lipoprotein lipase and its role in regulation of plasma lipoproteins and cardiac risk. Curr. Atheroscler. Rep..

[56] Khera A.V., Won H.H., Peloso G.M., O’Dushlaine C., Liu D., Stitziel N.O., Natarajan P., Nomura A., Emdin C.A., Gupta N. (2017). Association of rare and common variation in the lipoprotein lipase gene with coronary artery disease. JAMA.

[57] Yoshida K., Shimizugawa T., Ono M., Furukawa H. (2002). Angiopoietin-like protein 4 is a potent hyperlipidemia-inducing factor in mice and inhibitor of lipoprotein lipase. J. Lipid Res..

[58] Dewey F.E., Gusarova V., O’Dushlaine C., Gottesman O., Trejos J., Hunt C., Van Hout C.V., Habegger L., Buckler D., Lai K.M. (2016). Inactivating variants in ANGPTL4 and risk of coronary artery disease. N. Engl. J. Med..

[59] Clee S.M., Zhang H., Bissada N., Miao L., Ehrenborg E., Benlian P., Shen G.X., Angel A., LeBoeuf R.C., Hayden M.R. (1997). Relationship between lipoprotein lipase and high density lipoprotein cholesterol in mice: modulation by cholesteryl ester transfer protein and dietary status. J. Lipid Res..

[60] Willer C.J., Sanna S., Jackson A.U., Scuteri A., Bonnycastle L.L., Clarke R., Heath S.C., Timpson N.J., Najjar S.S., Stringham H.M. (2008). Newly identified loci that influence lipid concentrations and risk of coronary artery disease. Nat. Genet..

[61] Kathiresan S., Willer C.J., Peloso G.M., Demissie S., Musunuru K., Schadt E.E., Kaplan L., Bennett D., Li Y., Tanaka T. (2009). Common variants at 30 loci contribute to polygenic dyslipidemia. Nat. Genet..

[62] Holmes M.V., Asselbergs F.W., Palmer T.M., Drenos F., Lanktree M.B., Nelson C.P., Dale C.E., Padmanabhan S., Finan C., Swerdlow D.I. (2015). Mendelian randomization of blood lipids for coronary heart disease. Eur. Heart J..

[63] Di Angelantonio E., Sarwar N., Perry P., Kaptoge S., Ray K.K., Thompson A., Wood A.M., Lewington S., Sattar N., Emerging Risk Factors Collaboration (2009). Major lipids, apolipoproteins, and risk of vascular disease. JAMA.

[64] Whitlock G., Lewington S., Sherliker P., Clarke R., Emberson J., Halsey J., Qizilbash N., Collins R., Peto R., Prospective Studies Collaboration (2009). Body-mass index and cause-specific mortality in 900 000 adults: collaborative analyses of 57 prospective studies. Lancet.

[65] Nordestgaard B.G., Palmer T.M., Benn M., Zacho J., Tybjaerg-Hansen A., Davey Smith G., Timpson N.J. (2012). The effect of elevated body mass index on ischemic heart disease risk: causal estimates from a Mendelian randomisation approach. PLoS Med.

[66] Rao Kondapally Seshasai S., Kaptoge S., Thompson A., Di Angelantonio E., Gao P., Sarwar N., Whincup P.H., Mukamal K.J., Gillum R.F., Holme I. (2011). Diabetes mellitus, fasting glucose, and risk of cause-specific death. N. Engl. J. Med..

[67] Jansen H., Loley C., Lieb W., Pencina M.J., Nelson C.P., Kathiresan S., Peloso G.M., Voight B.F., Reilly M.P., Assimes T.L. (2015). Genetic variants primarily associated with type 2 diabetes are related to coronary artery disease risk. Atherosclerosis.

[68] Lewington S., Clarke R., Qizilbash N., Peto R., Collins R., Prospective Studies Collaboration (2002). Age-specific relevance of usual blood pressure to vascular mortality: a meta-analysis of individual data for one million adults in 61 prospective studies. Lancet.

[69] Lieb W., Jansen H., Loley C., Pencina M.J., Nelson C.P., Newton-Cheh C., Kathiresan S., Reilly M.P., Assimes T.L., Boerwinkle E. (2013). Genetic predisposition to higher blood pressure increases coronary artery disease risk. Hypertension.

[70] Harrison S.C., Holmes M.V., Burgess S., Asselbergs F.W., Jones G.T., Baas A.F., van ’t Hof F.N., de Bakker P.I.W., Blankensteijn J.D., Powell J.T. (2018). Genetic association of lipids and lipid drug targets with abdominal aortic aneurysm: a meta-analysis. JAMA Cardiol.

[71] Ference B.A., Ginsberg H.N., Graham I., Ray K.K., Packard C.J., Bruckert E., Hegele R.A., Krauss R.M., Raal F.J., Schunkert H. (2017). Low-density lipoproteins cause atherosclerotic cardiovascular disease. 1. Evidence from genetic, epidemiologic, and clinical studies. A consensus statement from the European Atherosclerosis Society Consensus Panel. Eur. Heart J..

[72] Rader D.J., Tall A.R. (2012). The not-so-simple HDL story: Is it time to revise the HDL cholesterol hypothesis?. Nat. Med..

[73] Pamir N., Pan C., Plubell D.L., Hutchins P.M., Tang C., Wimberger J., Irwin A., Vallim T.Q.A., Heinecke J.W., Lusis A.J. (2019). Genetic control of the mouse HDL proteome defines HDL traits, function, and heterogeneity. J. Lipid Res..

[74] Helgadottir A., Gretarsdottir S., Thorleifsson G., Hjartarson E., Sigurdsson A., Magnusdottir A., Jonasdottir A., Kristjansson H., Sulem P., Oddsson A. (2016). Variants with large effects on blood lipids and the role of cholesterol and triglycerides in coronary disease. Nat. Genet..

[75] Ference B.A., Kastelein J.J.P., Ray K.K., Ginsberg H.N., Chapman M.J., Packard C.J., Laufs U., Oliver-Williams C., Wood A.M., Butterworth A.S. (2019). Association of triglyceride-lowering LPL variants and LDL-C-lowering LDLR variants with risk of coronary heart disease. JAMA.

[76] Ginsberg H.N., Elam M.B., Lovato L.C., Crouse J.R., Leiter L.A., Linz P., Friedewald W.T., Buse J.B., Gerstein H.C., ACCORD Study Group (2010). Effects of combination lipid therapy in type 2 diabetes mellitus. N. Engl. J. Med..

[77] AIM-HIGH Investigators, Boden W.E., Probstfield J.L., Anderson T., Chaitman B.R., Desvignes-Nickens P., Koprowicz K., McBride R., Teo K., Weintraub W. (2011). Niacin in patients with low HDL cholesterol levels receiving intensive statin therapy. N. Engl. J. Med..

[78] Schwartz G.G., Olsson A.G., Abt M., Ballantyne C.M., Barter P.J., Brumm J., Chaitman B.R., Holme I.M., Kallend D., Leiter L.A. (2012). Effects of dalcetrapib in patients with a recent acute coronary syndrome. N. Engl. J. Med..

[79] Lincoff A.M., Nicholls S.J., Riesmeyer J.S., Barter P.J., Brewer H.B., Fox K.A.A., Gibson C.M., Granger C., Menon V., Montalescot G. (2017). Evacetrapib and cardiovascular outcomes in high-risk vascular disease. N. Engl. J. Med..

