# Supplementary material for: Lipid and metabolic syndrome traits in coronary artery disease: a Mendelian randomization study
Source: J Lipid Res. 2021 Feb 6;62:100044. doi: 10.1194/jlr.P120001000 (PMC7933489; doi:10.1194/jlr.P120001000)
Supplement: Data_Supplement [file mmc1.pdf]

Lipid and Metabolic Syndrome Traits in Coronary Artery Disease: A Mendelian Randomization Study

Data Supplement

Supplemental Figure S1. Comparison of lipid trait effects from Million Veteran Program and Global Lipid Genetics Consortium GWAS.

Supplemental Figure S2. Presence of residual pleiotropy in sets of lipid trait-restricted SNPs.

Supplemental Table S1. Genetic overlap at 185 lipid SNPs from Do, et al.

Supplemental Table S2. SNPs identified as pleiotropic outliers by MR-PRESSO.

Supplemental Table S3. Multivariable MR of lipid traits in CAD, including 8 SNPs identified as pleiotropic outliers by MR-PRESSO.

Supplemental Table S4. Multivariable MR of lipid and metabolic syndrome traits in CAD, including 3 SNPs identified as pleiotropic outliers by MR-PRESSO.

Supplemental Table S5. Lipid trait SNPs with large effect sizes.

Supplemental Table S6. Lipid and metabolic trait effect sizes for selected HDL-C-modifying loci.

Supplemental Table S7. Lipid and metabolic trait effect sizes for selected TG-modifying loci.

Supplemental Figure S1

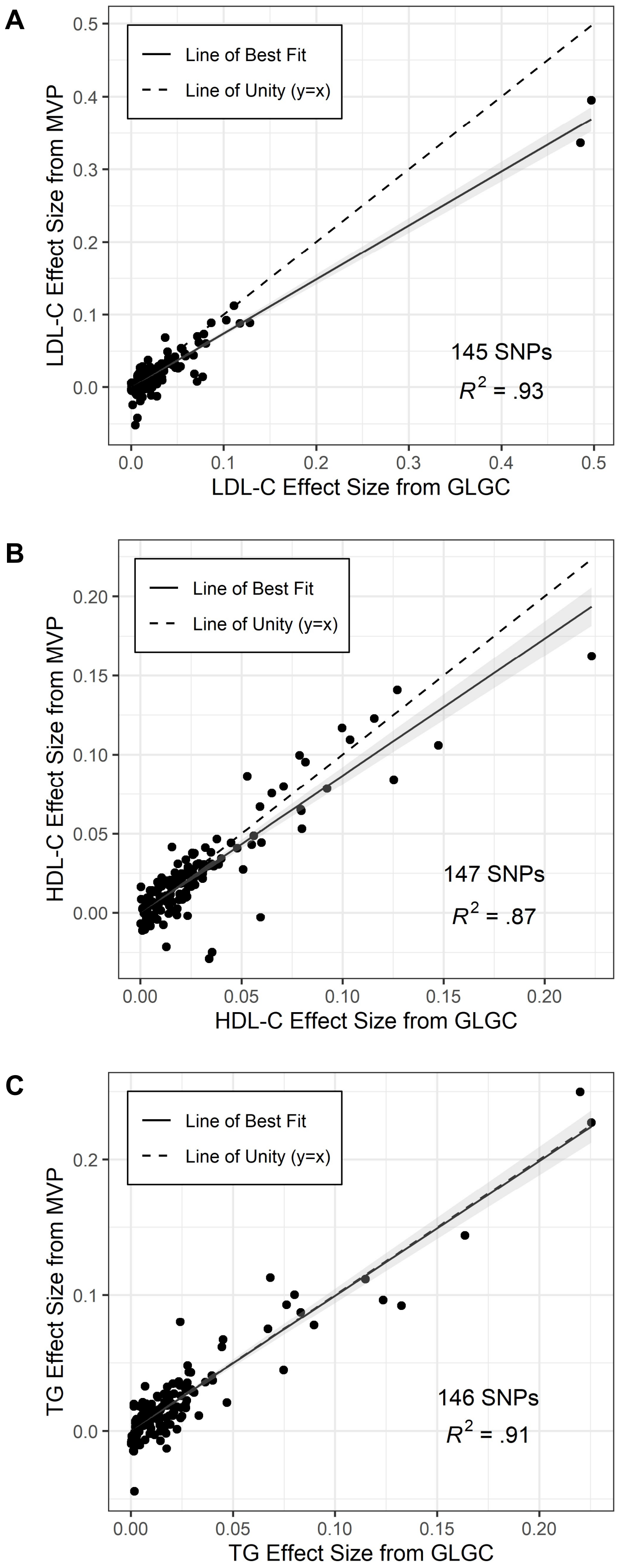

Supplemental Figure S1: Comparison of lipid trait effects from Million Veteran Program and Global Lipid Genetics Consortium GWAS

Points represent genome-wide significant lipid trait SNPs selected from the Million Veteran Program (MVP) GWAS where data from the Global Lipid Genetics Consortium (GLGC) GWAS was also available. Regression line (solid) and line of unity e.g. MVP estimate = GLGC estimate (dashed) are shown. Shaded (gray) region represents 95% confidence band for line of best fit. Deviation of solid line from dashed line represents attenuation of average effect estimate magnitude in the larger Million Veteran Program dataset. *(A-C)* Plot of LDL-C (A), HDL-C (B), or TG (C) effect size in s.d. units from GLGC against LDL-C (A), HDL-C (B), or TG (C) effect size in s.d. units from MVP.

Supplemental Figure S2

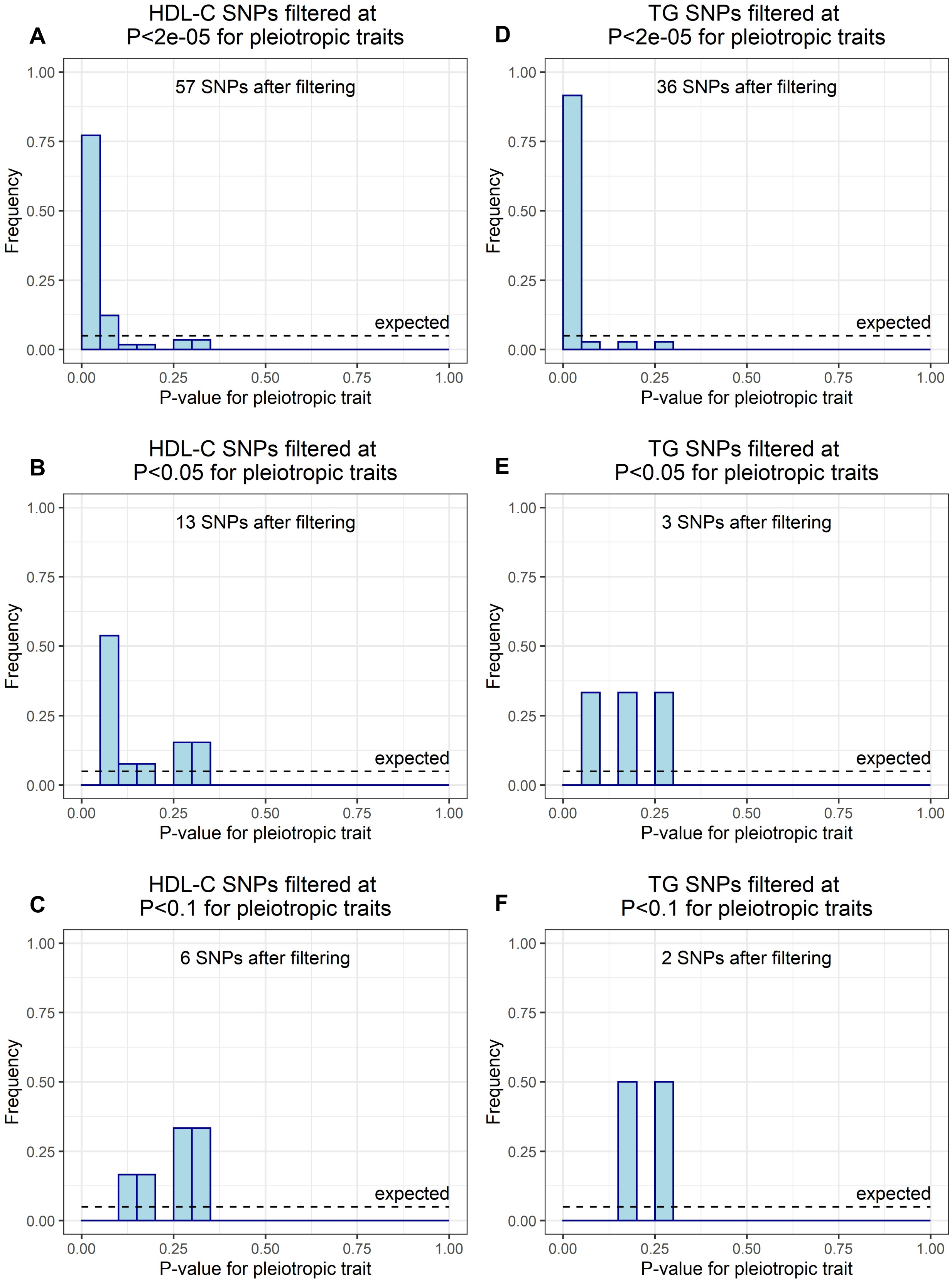

Supplemental Figure S2: Presence of residual pleiotropy in sets of lipid trait-restricted SNPs

Each plot represents a histogram of pleiotropic trait P-values for restricted sets of HDL-C and TG SNPs generated by filtering out SNPs with significant associations for other lipid and metabolic syndrome traits at the specified P-value threshold. P-value for pleiotropic trait represents the minimum (most significant) of other lipid and metabolic syndrome trait P-values for a given HDL-C or TG SNP. *(A-B)* Distributions of pleiotropic trait P-values for 57 HDL-C SNPs (A) or 36 TG SNPs (B) generated by filtering out pleiotropic SNPs at a Bonferroni-corrected P-value threshold of $P < 2 \times 10^{-5}$. *(C-D)* Distributions of pleiotropic trait P-values for 13 HDL-C SNPs (C) or 3 TG SNPs (D) generated by filtering out pleiotropic SNPs at a nominal P-value threshold of $P < 0.05$. *(E-F)* Distributions of pleiotropic trait P-values for 6 HDL-C SNPs (E) or 2 TG SNPs (F) generated by filtering out pleiotropic SNPs at a liberal P-value threshold of $P < 0.1$.

Supplemental Table S1. Genetic overlap at 185 lipid SNPs from Do, et al.

	Pleiotropic Trait						BMI,T2D, or SBP
Exposure	LDL-C	HDL-C	TG	BMI	T2D	SBP	
LDL-C	91%	30%	34%	18%	9%	16%	36%
HDL-C	21%	87%	51%	28%	22%	26%	47%
TG	40%	63%	90%	28%	22%	32%	52%

Percent of SNPs that are genome-wide significant ($P < 1 \times 10^{-8}$) for the exposure trait in Do, et al. with a secondary (pleiotropic trait) exposure that is significant after Bonferroni correction ($P < 2 \times 10^{-5}$). Diagonal entries for the lipid traits represent replication in the Million Veteran Program dataset.

Supplemental Table S2. SNPs identified as pleiotropic outliers by MR-PRESSO.

SNP	Outlier test $P < 0.05$ in:	Locus	LDL-C Beta	LDL-C P	HDL-C Beta	HDL-C P	TG Beta	TG P
rs186696265	LT MR<br>LTMS MR	*LPA*	0.186	4.2E-40	0.025	8.1E-02	-0.022	1.2E-01
rs10455872	LT MR<br>LTMS MR	*LPA*	-0.088	2.8E-54	-0.021	2.3E-04	0.013	2.3E-02
rs11751347	LT MR	*LPA*	-0.035	6.0E-11	-0.064	2.4E-32	0.037	4.2E-12
rs4977574	LT MR	9p21	0.017	2.3E-10	-0.001	6.3E-01	0.007	1.1E-02
rs653178	LT MR	*SH2B3*	0.026	3.7E-20	0.021	3.8E-14	-0.002	4.6E-01
rs2306363	LT MR	*MALAT1*	0.015	4.3E-05	0.023	4.6E-10	-0.017	2.3E-06
rs28394864	LT MR	*ZNF652*	0.0001	9.7E-01	-0.011	9.0E-05	0.022	8.0E-15
rs79949326	LT MR	*DAGLB*	0.011	1.2E-03	0.024	3.2E-13	0.002	6.4E-01
rs10811647	LTMS MR	9p21	0.011	7.2E-05	-0.005	1.1E-01	0.008	7.5E-03

SNP	BMI Beta	BMI P	T2D Beta	T2D P	SBP Beta	SBP P	CAD Beta	CAD P
rs186696265	-0.0016	8.5E-01	0.023	4.1E-01	0.525	1.1E-04	0.546	2.0E-97
rs10455872	0.0045	1.8E-01	-0.020	1.0E-01	-0.073	2.3E-01	-0.314	2E-153
rs11751347	-0.0004	9.0E-01	0.015	1.6E-01	-0.096	6.5E-02	-0.077	3.6E-14
rs4977574	0.0018	2.8E-01	-0.028	8.1E-06	-0.094	1.7E-03	-0.179	9E-223
rs653178	0.0091	1.3E-08	-0.021	1.5E-03	-0.576	6.8E-80	-0.055	1.1E-23
rs2306363	0.0021	3.8E-01	-0.040	3.8E-07	-0.436	5.2E-31	-0.041	4.9E-09
rs28394864	0.0082	1.8E-05	0.002	7.1E-01	0.244	5.6E-16	0.040	1.7E-12
rs79949326	0.0006	7.7E-01	-0.024	1.2E-03	-0.101	3.8E-03	-0.031	1.7E-06
rs10811647	0.0016	3.9E-01	-0.045	2.0E-12	-0.041	1.8E-01	-0.147	1E-159

LT MR: Lipid trait MR

LTMS MR: Lipid and metabolic syndrome trait MR

Beta: Effect size from GWAS association

Supplemental Table S3. Multivariable MR of lipid traits in CAD, including 8 SNPs identified as pleiotropic outliers by MR-PRESSO.

**Multivariable MR-IVW**						
	**OR**	**CI**	**P**	**I^2**		
**LDL-C**	1.60	1.47 - 1.74	1.8×10^{-23}	90%		
**HDL-C**	0.86	0.78 - 0.94	0.0013	90%		
**TG**	1.21	1.09 - 1.33	0.00022	90%		
**Multivariable MR-Egger**						
	**OR**	**CI**	**P**	**I^2**	**Int.**	**Int. P**
**LDL-C**	1.66	1.49 - 1.84	2.6×10^{-19}	90%	-0.0020	0.23
**HDL-C**	0.86	0.77 - 0.97	0.014	90%	-0.00027	0.88
**TG**	1.20	1.07 - 1.36	0.0024	90%	8.6×10^{-5}	0.96

Results of multivariable regression of CAD effect size on LDL-C, HDL-C, and TG effect size with the intercept fixed at zero (multivariable MR-IVW), or with estimation of the intercept (multivariable MR-Egger), including 8 pleiotropic SNPs identified by MR-PRESSO.

MR-IVW: Mendelian randomization, inverse-variance weighted

MR-Egger: Mendelian randomization-Egger regression

OR: odds ratio per unit change in exposure trait

CI: 95% confidence interval

Int.: MR-Egger Intercept

Supplemental Table S4. Multivariable MR of lipid and metabolic syndrome traits in CAD, including 3 SNPs identified as pleiotropic outliers by MR-PRESSO.

Multivariable MR-IVW						
	**OR**	**CI**	**P**	**I^2**		
**LDL-C**	1.56	1.47 - 1.65	3.6×10^{-50}	77%		
**HDL-C**	0.88	0.83 - 0.93	2.3×10^{-5}	77%		
**TG**	1.15	1.07 - 1.22	3.0×10^{-5}	77%		
**BMI**	1.32	1.23 - 1.41	8.5×10^{-16}	77%		
**T2D**	1.09	1.06 - 1.12	2.7×10^{-9}	77%		
**SBP**	1.038	1.034 - 1.042	1.8×10^{-73}	77%		
**Multivariable MR-Egger**						
	**OR**	**CI**	**P**	**I^2**	**Int.**	**Int. P**
**LDL-C**	1.66	1.56 - 1.76	2.1×10^{-54}	77%	-0.0019	1.6×10^{-6}
**HDL-C**	0.88	0.83 - 0.94	0.00025	77%	-0.00024	0.60
**TG**	1.17	1.10 - 1.26	6.3×10^{-6}	77%	-0.00078	0.084
**BMI**	1.47	1.33 - 1.62	7.0×10^{-14}	77%	-0.0016	0.0039
**T2D**	1.07	1.04 - 1.11	7.1×10^{-5}	77%	0.00090	0.090
**SBP**	1.036	1.031 - 1.042	1.1×10^{-38}	77%	0.00032	0.52

Results of multivariable regression of CAD effect size on LDL-C, HDL-C, TG, BMI, T2D, and SBP effect size with the intercept fixed at zero (multivariable MR-IVW), or with estimation of the intercept (multivariable MR-Egger), including 3 pleiotropic SNPs identified by MR-PRESSO.

MR-IVW: Mendelian randomization, inverse-variance weighted

MR-Egger: Mendelian randomization-Egger regression

OR: odds ratio per unit change in exposure trait

CI: 95% confidence interval

Int.: MR-Egger Intercept

Supplemental Table S5. Lipid trait SNPs with large effect sizes.

SNP	Trait with Large Effect:	Locus	LDL-C Beta	LDL-C P	CAD Beta	CAD P
rs118147862	LDL-C	*APOE-C1*	0.398	<1E-300	0.127	1.9E-16
rs11591147	LDL-C	*PCSK9*	0.395	3E-257	0.241	1.9E-22
rs7254892	LDL-C	*APOE-C1*	0.337	<1E-300	0.103	1.7E-10
rs143020224	LDL-C	*LDLR*	0.162	<1E-300	0.111	5.7E-34
rs77542162	LDL-C	*ABCA6*	0.162	4.0E-52	0.023	0.288
rs429358	LDL-C	*APOE-C1*	0.162	<1E-300	0.089	3.3E-27
**SNP**	**Trait with Large Effect:**	**Locus**	**HDL-C Beta**	**HDL-C P**	**CAD Beta**	**CAD P**
rs116843064	HDL-C	*ANGPTL4*	0.258	3E-137	-0.140	3.6E-10
rs268	HDL-C	*LPL*	0.241	2E-107	-0.127	4.4E-8
rs77960347	HDL-C	*LIPG*	0.233	2.9E-73	-0.104	8.8E-5
rs72836561	HDL-C	*CD300LG*	0.199	1E-123	-0.057	0.0022
rs143868709	HDL-C	*CETP*	0.182	2.0E-14	-0.041	0.37
rs117738782	HDL-C	*CETP*	0.173	7.2E-24	-0.012	0.72
rs1801177	HDL-C	*LPL*	0.162	3.1E-75	-0.130	4.9E-9
**SNP**	**Trait with Large Effect:**	**Locus**	**TG Beta**	**TG P**	**CAD Beta**	**CAD P**
rs116843064	TG	*ANGPTL4*	0.252	6E-134	0.140	3.6E-10
rs61905116	TG	*APOA1-C3*	0.250	<1E-300	0.059	7.2E-7
rs7350481	TG	*APOA1-C3*	0.227	<1E-300	0.056	1.0E-6
rs268	TG	*LPL*	0.217	4.8E-85	0.127	4.4E-8
rs72836561	TG	*CD300LG*	0.164	7.5E-84	0.057	0.0022

Beta: Effect size from GWAS association

Large effect: Beta > 0.15 for lipid trait (in units of s.d.)

Supplemental Table S6. Lipid and metabolic trait effect sizes for selected HDL-C-modifying loci.

SNP	Locus	LDL Beta	LDL P	HDL Beta	HDL P	TG Beta	TG P	BMI Beta	BMI P
rs77960347	*LIPG*	0.075	4E-09	0.233	3E-73	0.046	4E-04	-7E-04	0.93
rs17712538	*LIPG*	0.008	0.004	0.018	9E-11	0.001	0.75	-4E-04	0.80
rs2156552	*LIPG*	0.018	2E-06	0.065	4E-64	0.011	0.003	0.005	0.01
rs9958734	*LIPG*	0.024	2E-05	0.067	2E-32	0.009	0.10	-0.003	0.42
rs56070533	*LCAT*	0.002	0.55	0.078	9E-95	-0.009	0.02	-0.002	0.41
rs28578193	*LCAT*	-0.011	0.06	0.065	4E-28	-0.007	0.22	0.004	0.56
rs2218260	*LIPC*	0.019	4E-09	0.001	0.86	0.008	0.01	0.001	0.50
rs140525318	*LIPC*	-0.004	0.77	0.078	7E-11	-0.019	0.12	-0.004	0.45
rs261291	*LIPC*	0.006	0.03	0.092	4E-238	0.015	5E-08	0.001	0.60
rs1077834	*LIPC*	0.007	0.02	0.084	1E-172	0.021	5E-12	-0.002	0.27
rs11071371	*LIPC*	0.005	0.26	0.053	3E-36	0.013	0.003	-0.005	0.04
rs261337	*LIPC*	-0.004	0.39	0.030	8E-10	0.003	0.57	-0.003	0.34

SNP	Locus	T2D Beta	T2D P	SBP Beta	SBP P	CAD Beta	CAD P
rs77960347	*LIPG*	0.015	0.59	0.081	0.55	-0.104	9E-05
rs17712538	*LIPG*	0.017	0.009	-8E-04	0.98	-0.005	0.44
rs2156552	*LIPG*	0.010	0.24	-0.080	0.04	-0.010	0.16
rs9958734	*LIPG*	0.028	0.06	-0.052	0.48	-0.006	0.56
rs56070533	*LCAT*	0.001	0.89	0.034	0.47	0.019	0.03
rs28578193	*LCAT*	-0.049	0.20	-	-	0.017	0.46
rs2218260	*LIPC*	-0.014	0.04	0.033	0.28	-0.009	0.12
rs140525318	*LIPC*	-0.023	0.27	-	-	-0.004	0.85
rs261291	*LIPC*	-0.024	3E-04	0.045	0.16	0.019	0.001
rs1077834	*LIPC*	-0.005	0.56	0.011	0.76	0.025	5E-05
rs11071371	*LIPC*	-0.022	0.02	0.025	0.58	0.019	0.03
rs261337	*LIPC*	-6E-04	0.96	0.002	0.98	0.014	0.18

Beta: Effect size from GWAS association

- : SBP data not available (missing data)

Supplemental Table S7. Lipid and metabolic trait effect sizes for selected TG-modifying loci.

SNP	Locus	LDL Beta	LDL P	HDL Beta	HDL P	TG Beta	TG P	BMI Beta	BMI P
rs191430997	*LPL*	-0.026	0.07	-0.080	5E-08	0.096	5E-11	0.013	0.21
rs79821925	*LPL*	0.007	0.71	-0.107	1E-08	0.139	1E-13	-0.007	0.48
rs7003470	*LPL*	0.006	0.09	-0.015	3E-05	0.021	9E-09	0.003	0.18
rs1027265	*LPL*	0.004	0.30	-0.038	3E-18	0.048	1E-28	0.002	0.38
rs287	*LPL*	-0.006	0.05	-0.104	5E-260	0.118	<1E-300	0.006	0.005
rs268	*LPL*	-0.047	1E-05	-0.241	2E-107	0.217	5E-85	-0.004	0.53
rs16842	*LPL*	-0.011	3E-04	-0.024	8E-17	0.022	1E-14	-3E-04	0.88
rs1801177	*LPL*	-0.010	0.27	-0.162	3E-75	0.144	1E-58	0.002	0.80
rs34288216	*LPL*	-0.001	0.70	-0.029	5E-19	0.026	5E-15	-0.005	0.03
rs8107967	*ANGPTL4*	-0.002	0.45	-0.017	2E-10	0.018	1E-10	0.005	0.003
rs116843064	*ANGPTL4*	-0.054	1E-07	-0.258	3E-137	0.252	6E-134	-0.005	0.52
rs111500536	*ANGPTL4*	-0.007	0.13	-0.030	8E-11	0.038	2E-16	0.002	0.63
rs139340094	*MLXIPL*	-0.003	0.74	-0.025	0.02	0.061	3E-09	-0.002	0.68
rs13234131	*MLXIPL*	-0.017	7E-05	-0.036	2E-16	0.128	4E-191	-0.014	2E-06
rs62466267	*MLXIPL*	-0.008	0.16	-0.016	0.004	0.047	1E-17	-0.002	0.67
rs799157	*MLXIPL*	0.007	0.46	-0.038	2E-05	0.087	3E-22	-0.012	0.004
rs174537	*FADS1*	-0.028	3E-19	-0.031	2E-22	0.062	2E-86	0.003	0.09

SNP	Locus	T2D Beta	T2D P	SBP Beta	SBP P	CAD Beta	CAD P
rs191430997	*LPL*	-0.002	0.96	-	-	-9E-04	0.98
rs79821925	*LPL*	0.050	0.14	-	-	0.015	0.66
rs7003470	*LPL*	0.016	0.09	0.020	0.66	0.003	0.74
rs1027265	*LPL*	0.017	0.06	-0.005	0.92	0.016	0.05
rs287	*LPL*	0.037	6E-07	0.073	0.04	0.045	9E-12
rs268	*LPL*	0.031	0.20	0.047	0.68	0.127	4E-08
rs16842	*LPL*	-1E-04	0.99	0.013	0.70	0.017	0.002
rs1801177	*LPL*	0.065	0.01	0.067	0.58	0.130	5E-09
rs34288216	*LPL*	0.012	0.17	-3E-04	0.99	0.037	1E-06
rs8107967	*ANGPTL4*	0.044	1E-11	0.032	0.30	0.006	0.32
rs116843064	*ANGPTL4*	0.099	2E-05	0.374	0.001	0.140	4E-10
rs111500536	*ANGPTL4*	0.013	0.36	-0.100	0.14	0.043	2E-04
rs139340094	*MLXIPL*	-0.024	0.33	0.038	0.74	0.001	0.94
rs13234131	*MLXIPL*	-0.039	1E-04	0.094	0.04	0.008	0.36
rs62466267	*MLXIPL*	0.035	0.005	0.199	0.001	0.008	0.51
rs799157	*MLXIPL*	0.035	0.04	-	-	0.030	0.05
rs174537	*FADS1*	-0.028	3E-05	-0.050	0.12	-0.016	0.004

Beta: Effect size from GWAS association

- : SBP data not available (missing data)
